# Three-Dimensional-Printed Customized Orthodontic and Pedodontic Appliances: A Critical Review of a New Era for Treatment

**DOI:** 10.3390/children9081107

**Published:** 2022-07-23

**Authors:** Ioannis A. Tsolakis, Sotiria Gizani, Apostolos I. Tsolakis, Nearchos Panayi

**Affiliations:** 1 Department of Orthodontics, School of Dentistry, Aristotle University of Thessaloniki, Thessaloniki 54124, Greece; 2 Department of Paediatric Dentistry, Dental School, National and Kapodistrian University of Athens, Athens 11527, Greece; sotiriagizani@gmail.com; 3 Department of Orthodontics, National and Kapodistrian University of Athens, School of Dentistry, Athens 11527, Greece; apostso@otenet.gr; 4 Department of Orthodontics, C.W.R.U., Cleveland, OH 44106, USA; 5 Department of Orthodontics, European University of Cyprus, School of Dentistry, Egkomi 2404, Cyprus; dr.panayi@cytanet.com.cy; 6 School of Medicine, National and Kapodistrian University of Athens, Athens 15772, Greece

**Keywords:** 3D printing, CAD software, CoCr, resin, zirconia, customized orthodontic appliances

## Abstract

Three-dimensional (3D) designing and manufacturing technology is a direct derivative of digital technology. Three-dimensional volume and surface acquisition, CAD software, and 3D manufacturing are major changes included in daily practice in many orthodontic and pedodontic offices. Customized appliances can be designed using dental CAD software or general-purpose CAD software in the office or a laboratory. Materials that can be used are resins, alloys, or zirconia. Methods: The search strategy of this critical review included keywords in combination with MeSH terms in Medline, Scopus, and Cochrane Library up to June 2022 in the English language without any limit to the publication period. Results: According to our search, 12 articles were selected for our study. All the articles were in vitro prospective studies. Conclusions: The results suggested that almost all the known appliances can be designed and printed in a tailor-made fashion in contrast to the traditional one-size-fits-all approach. Customized appliances should be manufactured according to the patient’s needs, and this is justified by the certainty that this approach will be beneficial for the patient’s treatment. There is a need for more research on all direct 3D-printed appliances.

## 1. Introduction

In the last century, significant advancements have been introduced into our daily lives. Engineering, computers, and software are some of the significant parts of this evolution. Unavoidably, this change has affected medicine and dentistry, mainly in the aspects of diagnosis and treatment. In the last few years, advancements in 3D technology have allowed the three-dimensional (3D) designing and printing of customized orthodontic appliances [1]. Traditionally, almost all orthodontic appliances were designed and manufactured in the environment of an orthodontic laboratory. Dental arch impressions were taken and sent to the lab, where dental casts were poured using plaster. Depending on the kind of appliances to be made, acrylic, orthodontic laboratory wires, bands, soldering materials, premanufactured appliances parts, and wax were used. The appliances were in a way customized, meaning that they were made to fit the specific patient. Nevertheless, most appliance parts were bent and formed to match the particular patient’s needs. Most of the time, orthodontic laboratories were not installed in the area of the orthodontic office, but rather, in a private laboratory place.

**Figure 1:**
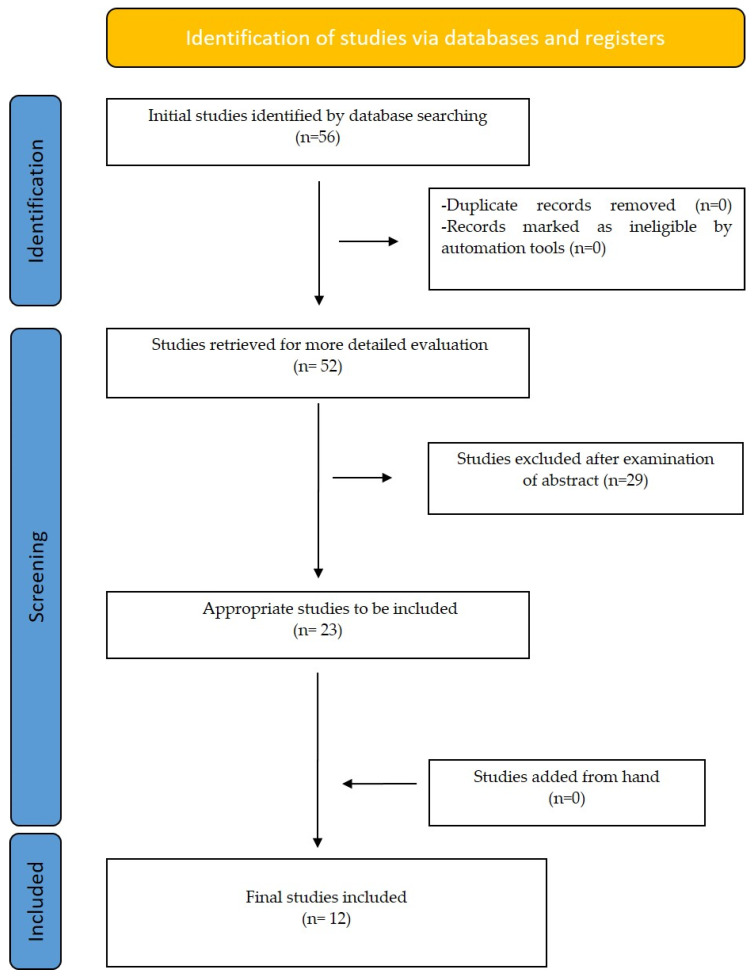
Prisma Flow diagram for selection of studies.

Nowadays, with the new digital technology advancements, many aspects of an orthodontic laboratory have moved into the space of the orthodontic office. The new environment of a modern laboratory has changed and somewhat moved into the screen of a computer. Using computer-aided design (CAD) software, the technician, dentist, or orthodontist can design various appliances, which can then be sent for 3D printing, either to 3D printers installed in the office, or outsourced in special laboratories where different kinds of 3D printers exist. The workflow of intraoral scanning, CAD software designing, and 3D printing has not only helped to decrease the number of materials for appliance manufacturing, the number of personnel required, and the space needed to build an orthodontic laboratory, but has also helped to reduce the burden of environmental sustainability due to less material being used for building appliances. A big advantage also is the ability to send the 3D file of the designed appliance via email to various laboratories worldwide for printing. A future project could use artificial intelligence (AI) software where big data from many offices could be gathered in special places where AI machines could analyze and send feedback to the orthodontist for future better treatments, suggestions on treatments, and growth predictions [1,2]. On the other hand, customized appliance manufacturing requires expensive machines and software such as scanners, CAD software, 3D printers, and post-printing units. There is also a considerable steep learning curve that the operator has to pass in order to be able to design and print the appliances. The cost of a printed customized appliance is somewhat higher than the traditional one. The materials for metal 3D printing are limited mainly to cobaltium-chromium (CoCr) and during the last year to stainless steel and titanium. Appliances such as occlusal splints, indirect bonding trays, printed aligners, and lately, customized orthodontic brackets, can be printed mainly using stereolithography (SLA) printers. SLA printers can be installed in the orthodontic office due to their small size and ease of use. In contrast, selective laser sintering (SLS) printers for metal printing (bed powder fusion technology) cannot be installed in the office due to their enormous size, the health hazards from materials used, and the post-printing procedure, which demands special machines [3]. Recently, a zirconia printer called Zipro (AON, Seoul, Korea) has been released in the market, allowing the manufacture of some appliances such as orthodontic brackets, lingual arches, and other space maintainers in the orthodontic office (Figure 1). Customized appliances made by metal printing are rigid and not flexible. For example, a customized band, compared to the traditional bands, cannot be bent and cannot be designed to pass the maximum circumference of the tooth. In addition, a metallic customized appliance cannot be made to exert force in a way that stainless steel could do. For this reason, a printed customized appliance often has to be combined with other non-printed parts, such as orthodontic wires, to create an active appliance (Table 1). Three-dimensional manufacturing is a general term for creating 3D objects which includes two types of manufacturing; subtractive manufacturing (milling) where the material is removed from a disc using computer-controlled tools, and additive manufacturing (3D printing) where the object is manufactured by laying down successive layers of material [3]. Milling is not used in creating orthodontic appliances mainly due to the excessive loss of material, the complexity of orthodontic appliances which cannot be easily manufactured this way, and the small thickness of the 20 mm discs which are mainly used to manufacture dental crowns and bridges. Nowadays, 3D printing is the main way to manufacture all kinds of orthodontic appliances. 

The introduction of three-dimensional printing technology to the dental field gave practitioners the ability to design and deliver low-cost customized appliances directly to the patients. This article aims to review the current literature on the use of 3D printed appliances and present the authors’ use of 3D technology in the orthodontic and pedodontic fields. 

## 2. Materials and Methods

This critical review was conducted by using the following keywords in the search strategy: “3D printing”, “orthodontics”, “paedodontics”, and “appliances”. These keywords were combined with the following Medical Subject Heading (MeSH terms): “Printing, Three-Dimensional” [Majr], “rapid prototyping” [MeSH Terms] “Orthodontic appliance” [Majr], “Orthodontics” [Majr], “Pedodontics” [Majr]. The databases used for the electronic search were Cochrane Library, Medline (PubMed), and Scopus. Additionally, a hand search was performed. The search was conducted for studies published up to June 2022. 

There was a selection of only English language articles without any limit to the publication period. The initial data search resulted in 56 studies. Out of all these papers, only 23 were selected by the abstract of the study. Each selected article was then fully evaluated by two different reviewers by reading the entire script. Finally, 12 papers were selected for the present critical review according to our inclusion and exclusion criteria (Table 2), (Figure 1).

## 3. Results

According to our search, 12 articles were selected for our study. All the articles were in vitro prospective studies. Three of these articles looked at the benefits of additive technology in dentistry. Two out of all of the articles studied the benefits of CAD/CAM technology. Three articles focused on the 3D printing fabrication of appliances that included bands. One introduced 3D printing of orthodontic brackets, and three looked at direct-printed clear aligners [4,5,6,7,8,9,10,11,12,13,14,15].

## 4. Additive Manufacturing (3D Printing)

Three-dimensional printing was invented in 1980 by Hideo Kodama of Nagoya Municipal Industrial Research Institute, Japan, who first presented the process for creating 3D plastic parts by photo-hardening polymers with UV exposure [16,17]. Later, in 1983, Charles (Chuck) Hull developed a system referred to as stereolithography, in which layers were added by curing photopolymers with ultraviolet (UV) lasers [18]. In 1986, Hull co-founded 3D Systems, Inc (Rock Hill, South Carolina, United States). to introduce the technology into the market, while the first-ever 3D printer, the SLA-1^®^, was introduced in November 1987.

The types of 3D printers that are mostly used in the area of dentistry are stereolithography and powder bed fusion printers. Stereolithography printers utilize UV light in order to cure photopolymers. Currently, there are three major kinds of printers; SLA, which uses a beam of laser to cure the photopolymer; DLP (direct light processing), which uses a light projector that projects slides of the 3D object to cure the resin, and MSLA (masked stereolithography), or LCD, which uses a panel of LCDs and a mask in order to allow the light needed to cure the resin [1,3,4,19,20,21,22,23,24]. Post-printing processing usually uses a washing machine where isopropyl alcohol 91% cleans the excess resin after 3D printing and a UV curing unit that cures the uncured part of the printed object. Powder bed fusion (SLS) printers are very big printers using a large laser tube in order to melt, join, or solidify metallic or plastic powder in order to create a 3D object. As mentioned before, those printers cannot be installed in an office. Units for sintering and debinding are needed, together with a gas supply to the printer′s chamber (argon), in order to remove oxygen, which creates problems in 3D printing. SLS printers mostly use CoCr powder for manufacturing customized appliances such as space maintainers, lingual arches, and RPEs. Stainless steel and titanium can also be used for printing in SLS printers [4,5,6].

## 5. CAD Software

CAD software is an essential part of 3D manufacturing. It has been in the market for the last 50 years mainly for aerospace and engineering. The purpose of CAD software is mostly to create, analyze, modify a design, and create a database for manufacturing. In recent years, orthodontic CAD software has been developed mainly for the design of orthodontic aligners, IDB trays, and splints, such as Maestro (Age solutions, Pisa, Italy), 3Shape appliance designer (Copenhagen, Denmark), and lately customized orthodontic brackets (Ubrackets, Coruo, Limoges, France). Some software, such as 3Shape appliance designer, can design appliances such as lingual arches, space maintainers, bands, and other various appliances which are mainly manufactured in metal. These software packages are dedicated to orthodontic appliance designing and include special tools to help the operator in designing. Nevertheless, designing appliances is also feasible with general-purpose CAD software, which can often be downloaded from the internet at no cost. A disadvantage of this CAD software is that the learning curve is quite steep and there are no special tools for appliance designing. Examples of this software are Meshmixer (Autodesk San Raphael, CA, USA), Blender (Amsterdam, Netherlands), and Rhinoceros (Seattle, WA 98103 USA). One way to learn designing is to watch tutorial videos on YouTube, which unavoidably requires a lot of time. Learning to design in such software gives more flexibility in designing where there are no limits in the 3D designing of an appliance [7,8]. 

## 6. Designing and Printing Workflow

**Figure 2:**
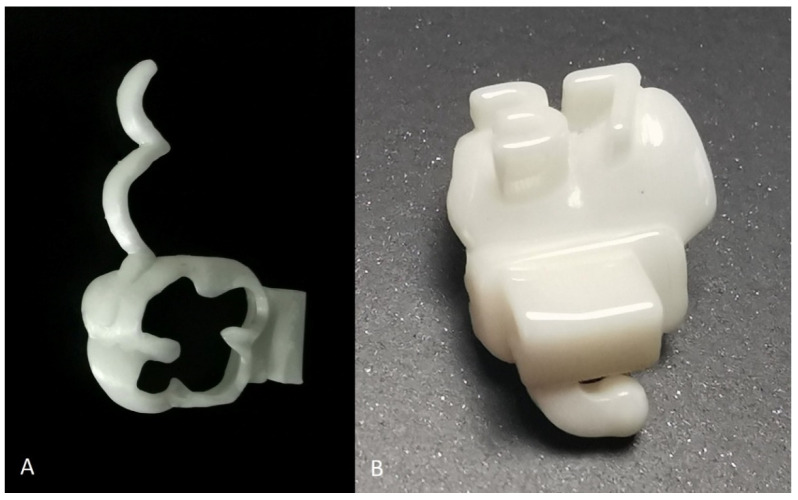
(**A**) Band and RPE arm made of printed zirconia (**B**) A customized tube with its positioning key and tooth number for accurate bonding was designed in Ubrackets CAD software.

The first procedure is the digitization of the dental arch by scanning using an intraoral scanner, or by scanning impressions or models using a desktop scanner. The next step is to import the dental scans into the CAD software for the appliance design. The software can be dedicated orthodontic CAD software or general-purpose CAD software. An example of designing a band using open-access software (Meshmixer) is shown in Figure 1. After designing the appliance, the 3D file, which is mostly in STL file format, is sent to a laboratory for undigitization. Undigitization is what we call the process of transforming a digital object into a real object using a form of 3D manufacturing. The appliance could be printed in CoCr alloy or other metal alloys. There is an option to print the object in zirconia by using the first zirconia printer, Zirpo (AON, Seoul, Korea). Zirconia printers can easily be installed in an office due to their compact size and the limited post-printing procedure. Nevertheless, metal printing, as mentioned before, cannot be performed in the office. After 3D printing, the appliance passes debinding, sintering, and polishing. The appliance is then ready for delivery [9,10,11] (Figure 2).

As a proof of concept, the authors designed bands that were printed in zirconia in a zirconia printer. Zirconia is a material of high hardness but low fracture toughness. For this reason, it is recommended to be used only in passive, non-exerting force appliances such as lingual arches and space maintainers. (Figure 2A).

## 7. Band Design

**Figure 3:**
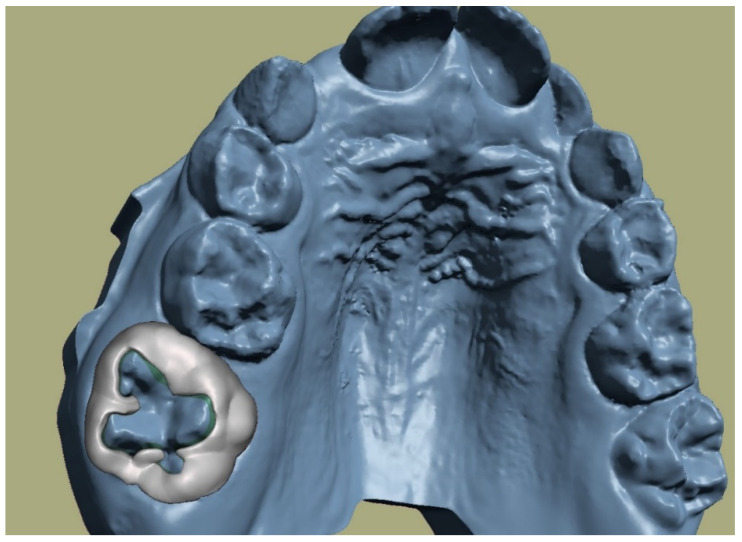
Designing a band in Meshmixer freeware. Standard band thickness 0.6 mm.

A band can be easily designed in an open-access software (Meshmixer)just by drawing the band around the tooth. Using more tools, the 2D drawing becomes a 3D object which can later be exported for printing. After an extensive design of customized appliances and bands, the authors propose a thickness of 0.6 to 0.7 mm, while the apical limits of the band should extend as apically as possible in order to ensure more retention. A helpful design is to extend arms from the band to the occlusal surface of the molar. Retention can be enhanced by designing a type of mesh at the inner part of the band. A band can be extended palatally and buccally to the adjacent teeth in cases where a rapid palatal expander (RPE) will be manufactured [9,10,11] (Figure 3).

## 8. Space Maintainer Appliances 

### 8.1. Lingual Arch Design

**Figure 4:**
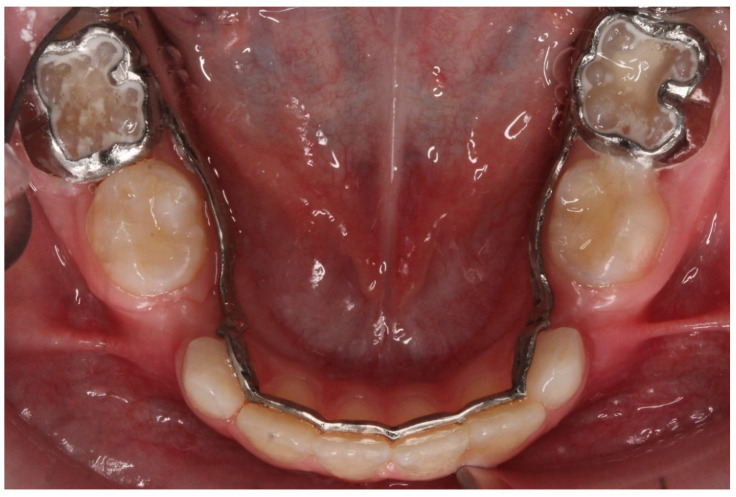
A lingual arch designed in Meshmixer and printed using CoCr alloy in SLS printer.

A lingual arch can easily be designed. It consists of two bands and an arch which is also designed by selecting the area on the anterior teeth where the arch will pass. Usually, the thickness of the arch is around 1 mm, and the height is 0.7 mm. A disadvantage of a customized lingual arch is that no loops can be positioned near the molar in case of a needed adjustment [11] (Figure 4).

### 8.2. Band and Loop Appliance

A unilateral space maintainer can easily be designed by combining a band and an archwire that lies on a tooth on the mesial side of the unerupted permanent tooth. 

### 8.3. Unerupted Molar Tooth Guiding Appliance

**Figure 5:**
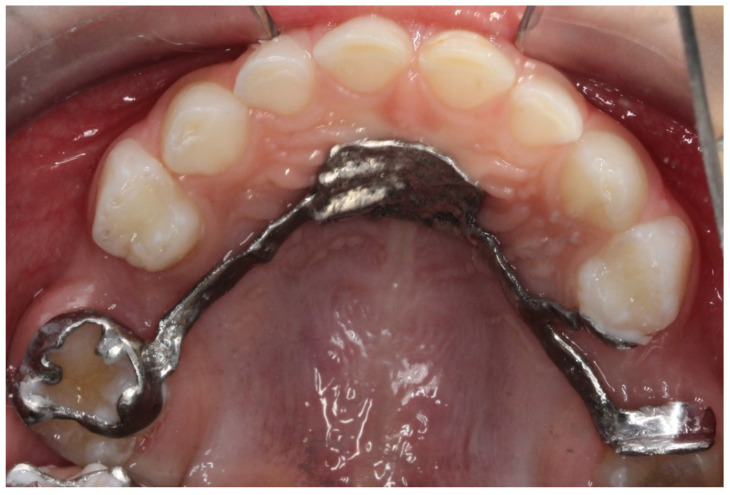
An unerupted molar eruption Nance hybrid guiding appliance. The appliance was inserted prior to molar eruption in order to guide its eruption.

In the case of a premature second deciduous molar loss, the first permanent molar most often tips anteriorly trying to find the distal part of the second deciduous molar in order to erupt. This creates problems for the molar itself, since after erupting it has to be orthodontically straightened, but also due to the loss of space for the eruption of the second premolar. For this reason, it is essential to design an appliance that will guide the molar to erupt in its position or prevent it from erupting mesially. In Figure 4, the molar tarted tipping anteriorly, closing the premolar space. For this reason, a Nance-like appliance with a guiding platform was designed to guide the molar into its place (Figure 5).

## 9. Rapid Palatal Expander (RPE) 

**Figure 6:**
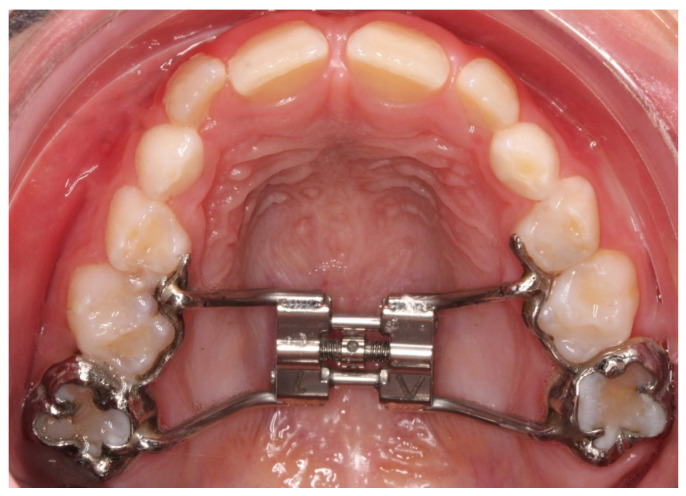
An RPE designed in Meshmixer and printed in CoCr. The screw is soldered to the customized bands on a printed dental model.

The only parts that can be designed in CAD software in an RPE case are the bands and the arms that extend mesially around the teeth. Expansion screws cannot be printed due to their complexity and high demand for manufacturing accuracy. RPEs can be designed in various ways; single bands, connected bands, bands with arms, or, in the case of a face mask, arms with hooks that can extend anteriorly for attaching elastics to the face mask [9,10]. The downside of these appliances is that they cannot be made in a one-step printing process (Figure 6).

## 10. Customized Brackets

**Figure 7:**
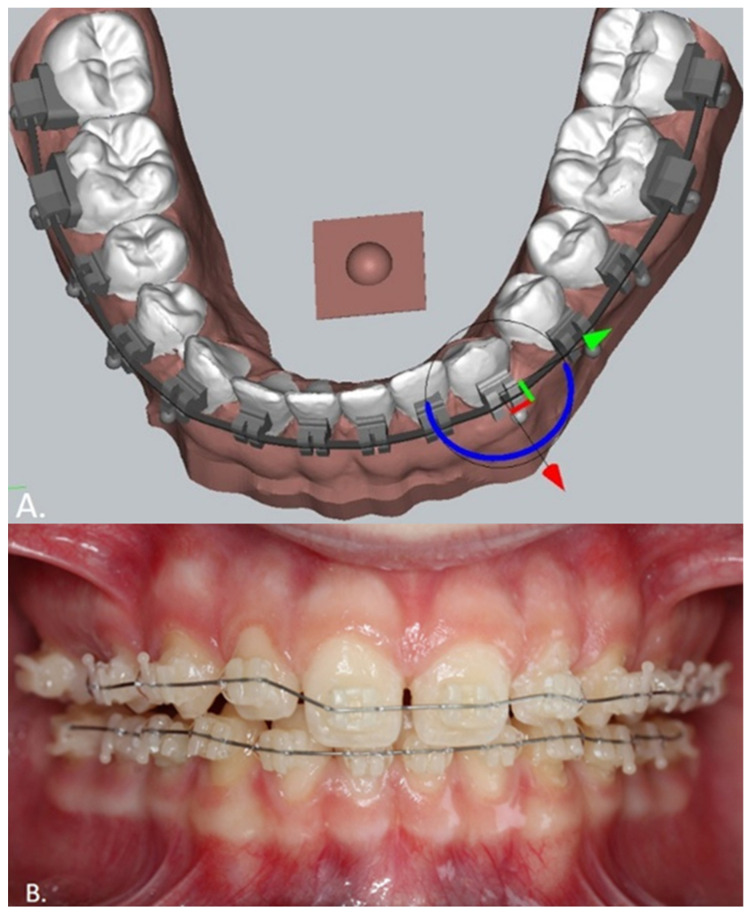
**(A**) Setup and automatic positioning of customized brackets in Ubrackets software. Setup is the first step in the customization process, followed by the automatic placement of the 0.018 × 0.025 inches slot brackets on a flat 0.018 × 0.025 inches archwire. (**B**) Customized brackets designed in Ubrackets software and printed in permanent crown resin by Bego (Bremen, Germany) bonded. The wire is a 0.012-inch Nitinol customized archwire bend on the exported pdf file archwire drawing.

CAD software has given practitioners the ability to individualize their orthodontic bracket prescription by direct-printed brackets. This is a promising advantage that could result in a more accurate and faster orthodontic treatment. A new software called Ubrackets, included in Deltaface software (Coruo, Limoges, France), has recently been released. The software enables the orthodontist to perform a setup while the software positions the virtual brackets on the teeth which are later printed in permanent crown resin or zirconia. Printed zirconia has high hardness, it is aesthetic, but has low fracture toughness. It is especially suitable for appliances that do not exert force on teeth (lingual arches, etc.). While the metals’ fracture toughness is proven, there are no studies that have looked at the fracture toughness of printed zirconia. This is the reason why we can only use zirconia for passive appliances such as lingual arches and nance appliances, but we cannot use it for rapid maxillary expanders and distalizing appliances. Zirconia can be painted after printing using special colors in order to match the patient’s tooth color. Each bracket is composed of a transfer key that can easily be removed after the bonding procedure is done [12] (Figure 7). The brackets can also be transferred to the mouth using an IDB tray designed in the Ubrackets software and printed using a special IDB tray resin. Permanent crown resin can also be used for bracket printing. Polymers have the advantage of high fracture toughness but low hardness and low elastic modulus. Low hardness causes wear of the bracket in cases of steel ligation or due to the harder archwire passing through the slot.

## 11. Clear Aligners

**Figure 8:**
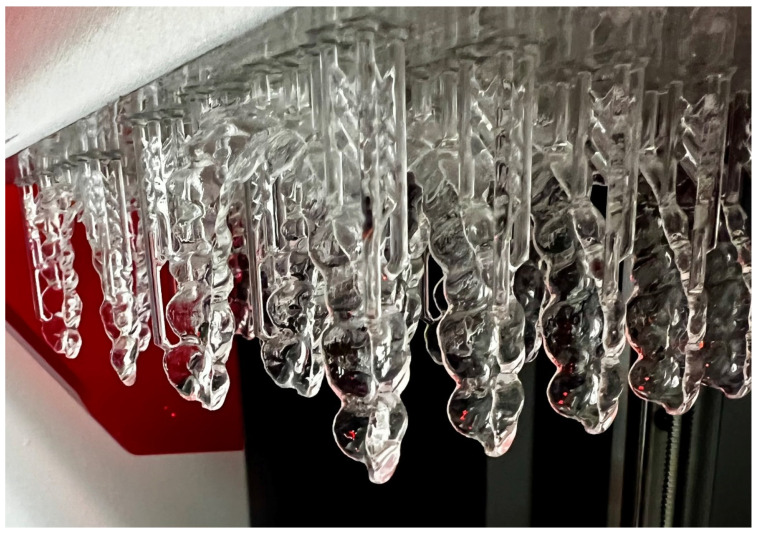
Direct-printed aligners.

By using CAD software, we can digitally move teeth to the final position and then create a set of multiple aligners. Those aligners will include only a short amount of movement each. Direct-printed aligners appear to have multiple advantages. One of the first materials that was used for direct-printed aligners or splints was Dental LT resin. Three-dimensional printed and suitably cured Dental LT resin-based clear dental aligners are suggested to be more suitable for patient use as they are geometrically more accurate. An aligner made from this resin shows an increased mechanical strength and elasticity in comparison to the conventionally produced thermoplastic-based thermoformed clear dental aligners. The direct-printed aligners have shown greater trueness and precision than thermoformed aligners. Later, another resin was introduced to the orthodontic field, TC-85 (Graphy, Seoul, Korea), which can constantly apply a light force to the teeth when used for the 3D printed clear aligners, owing to its flexibility and viscoelastic properties. In addition, the expected force decay induced by repeated insertion of the clear aligners is reduced and a constant orthodontic force can be maintained. Furthermore, its geometric stability at high temperatures and its shape memory properties provide advantages for clinical application [13,14,15] (Figure 8). Printed aligner manufacturing follows a specific protocol that should be accurately followed. Aligner designing CAD software is used to perform a digital setup while the aligners are drawn on the initial malocclusion model in the software. The software calculates the exact aligner number needed and exports them in STL file format. Printing is very important and can be performed using stereolithography printers. Graphy supplies the specific settings for each printer. The aligners can be placed perpendicularly, horizontally, or diagonally on the virtual software platform, while supports are essential for correct printing. After printing, the aligners are cleaned from the excess uncured resin using a centrifugation machine for 4 min followed by UV curing. UV curing results in a more complete polymerization of the aligners which gives them better mechanical properties and transparency. Graphy initially launched the Cure M curing unit which is more suitable for curing aligners. Recently Graphy released a new unit called Tera Harz which includes a nitrogen generator for better polymerization. Nitrogen is compressed into the unit chamber, removing the oxygen, resulting in a more complete polymerization. Supports are removed after curing of the aligners. The usual protocol is one aligner change per 7 to 14 days, depending on the doctor′s choice. More studies are being undertaken to study the properties of printed aligners and the behavior of aligners both in vivo and in vitro.

## 12. Discussion

Medicine and dentistry are sciences that in the last century have had tremendous advancement. Despite that, treatments have not been directed to the specific problems and characteristics of the patient, but rather have been applied in a one-treatment-fits-all concept. Nowadays, personalized or customized treatment has started to gain ground with the inclusion of digital technology in our lives. New diagnostic tools and machines, software, capturing devices, artificial intelligence, decoding of genes, new materials and medicine, and 3D printing have helped the initialization of concepts where the individual patient is at the core of the treatment. It appears that, in the near future and with the use of AI, prediction of growth will help design treatments that will have a more positive effect on the final treatment outcome. Automation, on the other hand, will help increase the speed of diagnosis and the accuracy of treatment prediction and appliance designing.

Customization in orthodontics began in the late 1990s when Weichmann invented the first customized lingual appliance. It was designed in CAD software and cast in gold, while the prototype archwires were bent by an archwire bending robot [25,26]. At the same time, intraoral scanning and cone beam computerized tomography (CBCT) started to appear. However, 3D printers were not well known in dentistry, and their price was quite high. A few years later more companies started designing and selling customized lingual appliances, while at the same time a company called Align introduced the first commercially available aligner system. Setup was performed digitally, and aligners were sent to the orthodontist for the aligner treatment [27,28]. Over time, software companies have developed CAD software for aligner treatments, IDB trays, splint designing, various appliance designing, clear aligners, and more recently, bracket designing. Due to the competition between the companies, printer prices have dropped, enabling more printers to be installed in private offices. Nevertheless, SLS printers for metal appliance printing are not available for office installation [29]. Printed zirconia appears to be a good alternative to CoCr, being resistant to fracture, more aesthetic, and hard, especially for appliances that do not exert force on teeth (RPEs, etc.). An advantage of zirconia printing is that zirconia printers can easily be installed in an office [30]. In dentistry, and especially in orthodontics, polymers are used in order to manufacture occlusal splints, IBD trays, dental models, permanent or temporary crowns, and printed aligners. Polymers are most often not used as functioning appliances due to their low hardness and elastic modulus. Low hardness means increased material wear, which in its turn affects the integrity of the appliance placed in the mouth [31]. However, recently polymer resins have been used to manufacture permanent or temporary crowns, inlays and onlays. The addition of ceramic nanoparticles reinforces the polymer by adding hardness and increasing fracture toughness. For this reason, permanent and temporary crown resins were tested for the 3D printing of customized orthodontic brackets [12]. Despite the fact that the permanent and temporary crown resins were not made for bracket printing, their hardness was almost double the value compared to the plastic brackets. Taking this into account, it seems that a possible enhancement of the properties of crown resins could lead to the development of a “bracket” resin for the in-house printing of customized brackets. Another way to maybe reinforce the mechanical properties of polymers for 3D printing is through the inclusion of fibers in the resin. According to an in vitro study, artificial saliva had no significant effect on fiber reinforced polymers mechanical properties even after 28 days [32]. Intraoral scanning has undoubtedly made the life of practitioners and patients much easier, due to the fast acquisition of the dental arches, ease of sharing data, and the absence of materials for taking impressions [33]. Intraoral scanning (surface scanning), in addition to the apparent advantage of digitizing dental arches, gives the dentist the ability to accurately capture the mouth of the patient, even in cases where traditional impressions would be difficult or even impossible to take. Patients with gag reflects can be a problem, while scanning is most of the time the solution. Newborn cleft patients can be scanned using small scanner tips, thus avoiding the dangerous alginate impressions which can be only taken in a hospital environment, often under sedation or general anesthesia [34]. However, at this point in time, materials to directly print NAM plates do not exist, therefore, the traditional way of manufacturing NAM plates follows scanning, dental model 3D printing, and then NAM manufacture. CAD software, on the other hand, enables the clinician to design the appliances on demand, whereas laboratories can manufacture the appliances faster with less material to use and with less personnel. The weaknesses of CoCr-printed appliances, as mentioned before, cannot be easily overcome. Nevertheless, printed appliances are a good alternative to traditional devices in cases where they can be used without creating problems. It is good to remember that we have to think about why we are doing something rather than simply doing something because we can. This does not mean that just because we can do something using technology, we have to do it. The justification for whether we have to use digital technology and customized appliances comes from the question: is this going to benefit the patients and their treatment?

## 13. Conclusions

Three-dimensional technology enables us to design and print customized appliances. Some of them can be designed and manufactured in the office; some must be printed in laboratories. Tailor-made appliances are a new approach to patient treatment that tends to overtake the traditional one-size-fits-all approach. Customized appliances should be manufactured according to the patient′s needs and justified by the certainty that this approach will be beneficial for the patient′s treatment. One of the strong advantages of 3D printing appliances is that they can be delivered on the same day to the patient’s mouth. There is a need for more research on all direct 3D-printed appliances. The weak link in printing customized orthodontic appliances is most probably materials. CAD software, printers and other units have been already developed to a satisfactory level. Materials, and specifically polymers, are what need to be optimized in order to be able to print appliances with excellent mechanical properties throughout all orthodontic treatment.

## Figures and Tables

**Table 1:** Three-dimensional printing materials used in Orthodontic and pedodontic appliances.

Materials	Characteristics	Use
Dental model resin	rigid, hard, high fracture toughness, temperature resistant	thermoforming procedure
Occlusal splint resin	transparent, medium fracture toughness	occlusal splints
IDB tray resin	transparent, soft	IDB tray
CoCr alloy	rigid, non-flexible, printed in SLS printers	metallic orthodontic appliances
Ti alloy	rigid, non-flexible, printed in SLS printers	metallic orthodontic appliances
Stainless steel alloy	rigid, non-flexible, printed in SLS printers	metallic orthodontic appliances
Permanent crown resin	low hardness, high fracture toughness	crowns, brackets (tested)
Zirconia slurry	high hardness, low fracture toughness, printed in zirconia printers	crowns, bridges, brackets, bands
Aligner resin	high elastic index, transparent, stable mechanical properties	printed aligners

**Table 2:** Inclusion and Exclusion criteria.

Inclusion Criteria	Exclusion Criteria
Studies that refer to 3D-printed customized orthodontic and pedodontic appliances.	Studies that are Reviews or authors′ opinion
In vitro studies prospective or retrospective.	

## Data Availability

Not applicable.
